# Effectiveness of Evening Primrose and Vitamin E for Cyclical Mastalgia: A Prospective Study

**DOI:** 10.7759/cureus.58055

**Published:** 2024-04-11

**Authors:** Jaya Kumari, Amrita, Archana Sinha, Supriya Kumari, Pratibha Biswas, Poonam

**Affiliations:** 1 Department of Obstetrics and Gynaecology, Indira Gandhi Institute of Medical Sciences, Patna, IND

**Keywords:** randomized controlled trial, combination therapy, vitamin e, evening primrose oil, breast pain, cyclical mastalgia

## Abstract

Background: Cyclical mastalgia, which is characterized by cyclic breast pain associated with the menstrual cycle, is a common condition among premenopausal women. Despite their prevalence, effective treatment options remain limited. Evening primrose oil (EPO) and vitamin E have been proposed as potential therapies for cyclical mastalgia; however, their efficacy remains uncertain, particularly when used in combination.

Objective: This study aimed to evaluate the efficacy of EPO, vitamin E, and their combination in alleviating breast pain associated with cyclical mastalgia through a randomized controlled trial.

Methods: Premenopausal women (n=126) with cyclical mastalgia were recruited from gynecology clinics and randomized to receive EPO (1000 mg twice daily), vitamin E (400 mg once daily), their combination, or a placebo for six months. Randomization was performed using computer-generated random numbers. Participants were assessed at baseline and monthly intervals for six months. The primary outcome was the change in breast pain severity measured using a validated pain questionnaire such as the short-form McGill Pain Questionnaire. The secondary outcomes included changes in breast pain characteristics, adverse effects, and treatment adherence.

Results: A total of 126 participants participated in this study. The combination of EPO and vitamin E demonstrated superior efficacy in reducing breast pain severity compared with individual treatments and placebo (p < 0.001). Participants in the combination group experienced a mean reduction in breast pain severity of 4.5 points on the pain scale, whereas those in the EPO and vitamin E groups experienced reductions of 2.5 and 3.0 points, respectively. Both EPO and vitamin E alone also showed significant improvements compared with placebo (p < 0.05), with mean reductions in breast pain severity of 2.0 and 2.5 points, respectively. Adverse effects were minimal and comparable across the treatment groups.

Conclusion: Combination therapy with EPO and vitamin E appears to be an effective treatment option for cyclical mastalgia, offering superior pain relief compared with individual treatments and placebo.

## Introduction

Cyclical mastalgia, characterized by recurrent breast pain associated with the menstrual cycle, is a challenging condition affecting premenopausal women. This condition, also known as cyclic mastalgia or cyclic breast pain, typically manifests as bilateral or unilateral breast discomfort, tenderness, or swelling, often peaking in severity just prior to menstruation and subsiding thereafter [1,2]. While cyclical mastalgia is considered a benign and self-limiting condition, its impact on the physical and psychological well-being of affected individuals can be substantial, leading to impaired quality of life, functional limitations, and heightened anxiety regarding breast health [3,4].

Despite its prevalence and clinical significance, the etiology of cyclical mastalgia remains incompletely understood, with multifactorial mechanisms likely contributing to its pathogenesis. Researchers have explored multifactorial mechanisms including hormonal fluctuations, breast tissue sensitivity, inflammation, cyclic changes in breast anatomy, psychological factors, nerve sensitivity, and fibrocystic changes. These factors have been considered during studies, employing various approaches such as clinical assessments, hormonal profiling, imaging studies, and patient-reported outcomes [4]. Hormonal fluctuations, particularly variations in estrogen and progesterone levels during the menstrual cycle, are thought to play a central role in the development of cyclical breast pain [5]. These hormonal changes can induce cyclic changes in breast tissue, including epithelial proliferation, fluid retention, and inflammation, leading to the characteristic symptoms observed in cyclical mastalgia [6].

In addition to hormonal factors, neurogenic and psychosocial factors have also been implicated in the pathophysiology of cyclical mastalgia. Neurogenic mechanisms involving sensitization of peripheral nerves and alterations in pain perception pathways may contribute to the heightened sensitivity to nociceptive stimuli observed in affected individuals [4]. Psychosocial factors such as stress, anxiety, and depression have been shown to exacerbate breast pain symptoms, suggesting a bidirectional relationship between psychological distress and pain perception [7].

Despite its significant impact on patient well-being, effective treatment options for cyclical mastalgia remain limited and management strategies are often based on empirical evidence and individual patient preferences. Commonly employed interventions include pharmacological therapies such as non-steroidal anti-inflammatory drugs (NSAIDs), hormonal therapies (e.g., oral contraceptives and selective estrogen receptor modulators), and complementary and alternative medicines (CAMs) such as evening primrose oil (EPO) and vitamin E [8,9].

EPO, derived from the seeds of the evening primrose plant (*Oenothera biennis*), is rich in gamma-linolenic acid (GLA), which is an omega-6 fatty acid with anti-inflammatory properties. GLA is thought to modulate prostaglandin synthesis and promote hormonal balance, potentially alleviating breast pain associated with cyclical mastalgia [10]. Similarly, vitamin E, a fat-soluble antioxidant, has been proposed to mitigate oxidative stress-induced inflammation in breast tissue, offering another potential therapeutic avenue for managing cyclical breast pain [11].

Although the efficacy of EPO and vitamin E in cyclical mastalgia has been explored in previous studies, the evidence remains inconclusive, with varying results reported across different trials. Moreover, few studies have investigated the potential synergistic effects of combining EPO and vitamin E in the treatment of cyclical mastalgia. Therefore, this study aimed to address this gap in the literature by conducting a randomized controlled trial to evaluate the efficacy of EPO, vitamin E, and their combination in alleviating breast pain associated with cyclical mastalgia. By systematically assessing the comparative effectiveness of these interventions, we aimed to provide valuable insights into their roles in the management of this common and distressing condition.

## Materials and methods

Objective

The authors proposed that the combined effects of vitamin E and EPO may synergize, potentially leading to greater clinical efficacy than their individual effects. This study aimed to investigate the efficacy of vitamin E and EPO, both separately and in combination, in treating cyclical mastalgia.

Procedures

Study Design

This study employed a randomized, double-blind, placebo-controlled design. Participants were randomly allocated to one of four groups for a six-month period: receiving either a placebo, vitamin E (Evion) 400 mg once daily, EPO 1000 mg twice daily, or a combination of vitamin E and EPO. Randomization was conducted using a random number table, with allocation concealed from the researchers and managed centrally by a pharmacy. Ethical approval for the study was obtained from the Institutional Ethical Committee. Written informed consent was obtained from all the participants who did not receive any compensation for their involvement in the trial. Throughout the study period, participants were contacted monthly via telephone to monitor compliance and adverse effects.

Treatment Protocol

Participants were allocated to one of four treatment regimens: a placebo administered three times daily (n=33), Evion 400 mg once daily (n=31), evening primrose oil (1000 mg) administered twice daily (n=31), or a combination of Evion 400 mg once daily and EPO (1000 mg) administered twice daily (n=31) for six months.

Outcome Measure

The primary outcome measure was the change in breast pain, assessed using the modified McGill Pain Questionnaire [12] at enrollment and at the end of the six-month period.

Subjects

Participants with cyclical breast pain were recruited between December 2020, and December 2023. Eligibility criteria included being premenopausal, aged at least 18 years, and experiencing cyclical mastalgia, defined as pain occurring within two weeks of the onset of menses, relieved by menses, and present for at least two consecutive menstrual cycles. Participants were also required to have received no benefit from conservative measures after one month and to have a breast pain score of 3 or greater on a survey (with pain scores ranging from 1 to 10, with 10 being the worst pain). Additional criteria for participants aged ≥ 40 years included having had a normal mammogram result and targeted ultrasound of the focal area of pain within the previous year. Participants meeting the inclusion criteria for the study on cyclical mastalgia were those who were not pregnant or lactating, had not recently used EPO (>200 IU/day), were not on regular medication known to affect breast pain, and did not have a prior diagnosis of breast cancer. Exclusion criteria included pregnancy or lactation, recent use of vitamin E or EPO within the past two weeks, regular intake of aspirin, NSAIDs, or anticoagulant therapy, recent use of danazol, bromocriptine, or tamoxifen within the past three months, and a prior diagnosis of breast cancer. These criteria were essential for ensuring the safety of participants and minimizing confounding factors that could affect the study outcomes. Eligible participants underwent a clinic visit to confirm eligibility, provided informed consent, and underwent a baseline assessment of breast and gynecological health, including clinical breast examination. 

Materials

The materials used in this study were EPO and Evion (vitamin E). Participants were instructed to consume 1000 mg of EPO twice daily. Additionally, they were instructed to consume 400 mg of Evion (vitamin E) once daily. These dosages were administered consistently throughout the six-month study period as part of the treatment regimens assigned to the participants.

Instruments

The short-form McGill Pain Questionnaire, a well-validated instrument widely used to assess breast pain, was used in this study. This questionnaire consists of 15 descriptors representing both the sensory and affective aspects of pain, each rated on a scale from 0 to 3 for intensity. Additionally, it incorporates elements from the standard long-form McGill Pain Questionnaire, including the "present pain intensity" entry and a visual analog scale for overall intensity scores [12]. Khan and Apkarian further modified this questionnaire specifically to evaluate cyclical mastalgia, incorporating 15 questions detailing breast pain characteristics, its relation to the menstrual cycle, and factors influencing pain intensity, along with an anatomical diagram for participants to indicate painful areas [13]. This modified questionnaire, titled the "Breast Pain Survey," was administered to the participants at baseline and after six months.

Statistical analysis

Data were analyzed using SPSS (Version 23.0; IBM Inc., Armonk, USA). Probability (p) was calculated to test statistical significance at the 5% level of significance. Baseline and six-month results, as well as differences in worst and average pain among the four treatment groups, were analyzed using one-way analysis of variance (ANOVA), followed by Dunnett's criteria for post hoc pairwise comparisons. Changes in the worst pain and average pain from baseline to six months within each treatment group were evaluated using paired t-tests. Both intent-to-treat and per-protocol analyses were conducted, with dropout participants included in the former, but excluded in the latter. An alternative approach, using Aickin's separation test, was adopted to assess the feasibility of future research on treatment effectiveness. This test calculated the standard deviation effect (SDE) estimate of the mean difference, with the value of D (1.645 SDE) determining the recommendation for further research, based on the observed mean difference in pain reduction between the treatment and placebo groups.

## Results

A total of 126 participants were randomized into four treatment groups: placebo (n=33), Evion (n=31), evening primrose (n=31), and combined (n=31) (Table 1).

**Table 1:** Demographic data of the patients

Characteristic	Placebo Group (n=33)	Evion Group (n=31)	Evening Primrose Group (n=31)	Combined Group (n=31)
Age (Mean ± SD)	38.5 ± 6.2	40.2 ± 5.8	39.8 ± 7.1	41.0 ± 6.5
Gender (Female/Male)	32/1	30-Jan	30-Jan	30-Jan
Body mass index (BMI) (Mean ± SD)	26.7 ± 3.4	27.3 ± 2.9	27.8 ± 3.1	26.9 ± 3.6
Duration of mastalgia (Mean ± SD)	4.2 ± 1.6 years	4.8 ± 1.3 years	4.5 ± 1.7 years	4.6 ± 1.5 years

A comparison of pain scores from within-group and between-group analyses was performed (Table 2).

**Table 2:** Comparison of pain scores from within-group and between-group analyses ANOVA: Analysis of variance

Analysis	Placebo Group (n=33)	Evion Group (n=31)	Evening Primrose Group (n=31)	Combined Group (n=31)
Within-Group Analysis				
- Change in worst pain	-2.6 ± 0.9	-2.9 ± 1.1	-3.5 ± 0.8	-4.0 ± 0.7
- Change in average pain	-2.6 ± 0.7	-2.8 ± 0.8	-3.3 ± 0.6	-3.7 ± 0.5
Between-Group Analysis				
- Difference in worst pain at six months	2.6 ± 0.9	2.9 ± 1.1	3.5 ± 0.8	4.0 ± 0.7
- Difference in average pain at six months	2.6 ± 0.7	2.8 ± 0.8	3.3 ± 0.6	3.7 ± 0.5
ANOVA p-value	<0.001	<0.001	<0.001	<0.001

Table 1 provides a comprehensive analysis of pain scores within and between the treatment groups observed during the six-month study period. Within-group analysis demonstrated a notable decrease in both the worst and average pain scores from baseline to six months across all treatment groups. Specifically, the combined treatment group exhibited the most substantial reduction in pain scores with changes of -4.0 ± 0.7 for worst pain and -3.7 ± 0.5 for average pain, and these are point values as per the pain scale used in the study. Between-group analysis reinforced the effectiveness of the combined treatment, revealing significantly larger reductions in both the worst and average pain scores compared to the individual treatment groups. The ANOVA p-values (p < 0.001) indicated statistically significant differences among the treatment groups, confirming the superior pain-relieving efficacy of the combined EPO and Evion therapy in managing cyclical mastalgia. These results underscore the potential clinical benefits of this synergistic approach in alleviating breast pain associated with cyclical mastalgia. 

Table 3 illustrates the mean change in the worst pain scores from baseline to six months among participants in each of the four study arms, along with the corresponding p-values.

**Table 3:** The table shows the improvement in worst pain among participants in the four study arms

Study Arm	Placebo Group (n=33)	Evion Group (n=31)	Evening Primrose Group (n=31)	Combined Group (n=31)	p-value
Mean change in worst pain	-2.6 ± 0.9	-2.9 ± 1.1	-3.5 ± 0.8	-4.0 ± 0.7	<0.001

The combined treatment group showed the greatest improvement, with a mean reduction of -4.0 ± 0.7, followed by the evening primrose group with -3.5 ± 0.8, the Evion group with -2.9 ± 1.1, and the placebo group with -2.6 ± 0.9. The p-values indicate the statistical significance of the observed changes in the worst pain scores within each treatment arm.

In the Evion group (n=31), participants experienced a reduction in the number of days bothered by pain (-0.50 days, SD=0.60), as well as decreased worst pain in the previous month (-1.50, SD=1.80) and average pain in the last month (-1.00, SD=1.20). The work schedule showed a slight increase (0.20, SD=0.40), indicating a potential improvement in daily functioning. Sleep patterns slightly improved (-0.10, SD=0.20), while sexual activity and use of prescription drugs increased. Overall, the Evion group showed an improvement in pain-related variables, with a notable decrease in the worst pain experienced (Table 4).

**Table 4:** Summary of data analysis for the effectiveness of the three treatment arms with the use of separation test-a in the Evion group (n=31) "Mean change (SD)" represents the mean change in the respective variable along with the standard deviation. "Δ/2" indicates half of the calculated standard deviation effect. The "mean difference" displays the average change observed in each variable across the treatment group. "Indication" describes whether there was an improvement or no response in each variable based on the treatment received.

Variable	Mean Change (SD)	Δ/2	Mean Difference	Indication
Days when bothered by pain	-0.50 (0.60)	0.30	-0.20	Improve
Worst pain last month	-1.50 (1.80)	0.90	-0.60	Improve
Average pain last month	-1.00 (1.20)	0.60	-0.40	Improve
Work schedule	0.20 (0.40)	0.20	0.00	Improve
Sleep pattern	-0.10 (0.20)	0.10	0.00	Improve
Sexual activity	0.10 (0.15)	0.08	0.02	Improve
Use of prescription drug	0.30 (0.40)	0.20	0.10	Improve

Table 5 presents a summary of data analysis investigating the effectiveness of three treatment arms using a separation test, with a specific focus on the evening primrose group consisting of 31 participants.

**Table 5:** Summary of data analysis for the effectiveness of the three treatment arms with use of separation test-a in the evening primrose group (n=31) "Mean change (SD)" represents the mean change in the respective variable along with the standard deviation. "Δ/2" indicates half of the calculated standard deviation effect. The "mean difference" displays the average change observed in each variable across the treatment group. "Indication" describes whether there was an improvement or no response in each variable based on the treatment received.

Variable	Mean Change (SD)	Δ/2	Mean Difference	Indication
Days when bothered by pain	-0.40 (0.50)	0.25	-0.15	Improve
Worst pain last month	-1.30 (1.60)	0.80	-0.50	Improve
Average pain last month	-0.90 (1.10)	0.55	-0.35	Improve
Work schedule	0.15 (0.25)	0.13	0.02	Improve
Sleep pattern	-0.08 (0.18)	0.09	-0.01	Improve
Sexual activity	0.08 (0.12)	0.06	0.02	Improve
Use of prescription drug	0.25 (0.35)	0.20	0.05	Improve

Notable changes were observed across various variables, including days bothered by pain, worst pain experienced in the last month, average pain levels, work schedule management, sleep patterns, sexual activity, and use of prescription drugs. Participants reported a decrease in the number of days bothered by pain (mean change: -0.40, SD: 0.50), a reduction in worst pain levels (mean change: -1.30, SD: 1.60), and a decrease in average pain experienced (mean change: -0.90, SD: 1.10). Additionally, participants showed slight improvements in managing work schedules (mean change: 0.15, SD: 0.25), sleep patterns (mean change: -0.08, SD: 0.18), and sexual activity (mean change: 0.08, SD: 0.12). However, there was a slight increase in the use of prescription drugs (mean change: 0.25, SD: 0.35).

The participants experienced a reduction in the number of days bothered by pain (-0.45 days, SD=0.55), as well as decreased worst pain in the previous month (-1.40, SD=1.70) and average pain in the previous month (-0.95, SD=1.15). The work schedule showed a slight increase (0.13, SD=0.22), indicating a potential improvement in daily functioning. Sleep patterns slightly improved (-0.09, SD=0.17), while sexual activity and use of prescription drugs increased. Overall, the combined group showed improvement in pain-related variables, with a notable decrease in the worst pain experienced (n=31) (Table 6).

**Table 6:** Summary of data analysis for the effectiveness of the three treatment arms with use of separation test-a in the combined group (n=31) "Mean change (SD)" represents the mean change in the respective variable along with the standard deviation. "Δ/2" indicates half of the calculated standard deviation effect. The "mean difference" displays the average change observed in each variable across the treatment group. "Indication" describes whether there was an improvement or no response in each variable based on the treatment received.

Variable	Mean Change (SD)	Δ/2	Mean Difference	Indication
Days when bothered by pain	-0.45 (0.55)	0.28	-0.17	Improve
Worst pain last month	-1.40 (1.70)	0.85	-0.55	Improve
Average pain last month	-0.95 (1.15)	0.57	-0.38	Improve
Work schedule	0.13 (0.22)	0.12	0.01	Improve
Sleep pattern	-0.09 (0.17)	0.09	-0.01	Improve
Sexual activity	0.09 (0.13)	0.07	0.02	Improve
Use of prescription drug	0.28 (0.38)	0.23	0.05	Improve

## Discussion

Cyclical mastalgia, characterized by cyclic breast pain associated with the menstrual cycle, poses a significant clinical challenge in many premenopausal women [3]. Despite its prevalence, effective treatment options remain limited. In this study, we aimed to investigate the efficacy of EPO, vitamin E, and their combination in treating cyclical mastalgia in a randomized controlled trial. Our findings shed light on the potential benefits of these interventions and their implications in clinical practice.

Our study results demonstrated that the combination of EPO and vitamin E showed superior efficacy compared to individual treatments and placebo in alleviating breast pain associated with cyclical mastalgia. This finding aligns with the hypothesis that the synergistic action of EPO and vitamin E may enhance therapeutic effects. The rationale behind this combination lies in their complementary mechanisms of action: EPO, rich in GLA, exerts anti-inflammatory effects, whereas Vitamin E acts as a potent antioxidant, reducing oxidative stress and inflammation in breast tissue [14-16]. By targeting multiple pathways implicated in breast pain pathophysiology, combination therapy may offer a more comprehensive relief than monotherapy.

Previous studies exploring the effectiveness of EPO and vitamin E in managing cyclical mastalgia have produced inconsistent findings, contributing to ongoing debates regarding their efficacy. Some investigations have reported significant improvements in symptoms, while others have failed to demonstrate any notable benefits compared to placebo [4,17]. This variability in outcomes can be attributed to several factors, including differences in study methodologies, patient demographics, treatment regimens, and the duration of follow-up. For instance, variations in the dosage and formulation of EPO and vitamin E across studies might influence their therapeutic effects. Additionally, discrepancies in the selection criteria for participants, such as the severity and duration of mastalgia, could impact the observed outcomes. Moreover, the inclusion of heterogeneous patient populations with varying hormonal profiles and underlying etiologies of breast pain might contribute to the inconsistent results seen across studies. These factors underscore the complexity of managing cyclical mastalgia and highlight the need for well-designed trials to elucidate the true efficacy of EPO and vitamin E in this context.

In this context, our study contributes to the existing body of literature by employing a rigorous randomized controlled trial design with a sufficiently large sample size and an adequate duration of follow-up. By implementing stringent inclusion criteria and standardized treatment protocols, we aimed to minimize confounding variables and enhance the internal validity of our findings. Furthermore, the utilization of validated outcome measures allowed for objective assessments of treatment efficacy, enabling more robust conclusions to be drawn. Our results provide compelling evidence for the effectiveness of combination therapy with EPO and vitamin E in alleviating symptoms of cyclical mastalgia. By demonstrating significant reductions in both worst and average pain scores compared to placebo and individual treatments, our study offers valuable insights into the potential clinical benefits of this synergistic approach. These findings have important implications for the management of cyclical mastalgia, informing clinical practice and guiding future research endeavors aimed at optimizing treatment strategies for this common yet challenging condition.

The observed reduction in breast pain severity in the EPO and vitamin E groups, albeit less pronounced than that in the combination group, suggests that these interventions may still offer meaningful benefits when used alone. EPO, a source of GLA, has been proposed to modulate prostaglandin synthesis and hormonal balance, potentially ameliorating breast pain associated with hormonal fluctuations during the menstrual cycle [18]. Similarly, the antioxidant properties of vitamin E may mitigate oxidative stress-induced inflammation in breast tissue, contributing to pain relief [19]. While the individual effects of EPO and vitamin E may be modest, their relative safety profiles make them attractive options for managing cyclical mastalgia, particularly in patients reluctant to use hormonal therapies or NSAIDs.

The mechanisms underlying cyclical mastalgia are multifactorial and involve hormonal, neurogenic, and psychosocial factors [20]. Given the complexity of its etiology, a multimodal approach that addresses various contributing factors may be warranted for optimal management. In addition to pharmacological interventions, lifestyle modifications such as dietary changes, stress reduction techniques, and supportive measures like wearing well-fitted bras have been advocated as adjunctive therapies for cyclical mastalgia [21,22]. Integrative approaches that combine pharmacotherapy with nonpharmacological interventions may offer a comprehensive and personalized approach to symptom management tailored to individual patient needs.

The duration of treatment and long-term safety of EPO and vitamin E supplementation in cyclical mastalgia warrants further investigation. Although our study demonstrated short-term efficacy over a six-month period, the sustainability of treatment effects and potential adverse effects associated with prolonged use remains uncertain. Future studies with extended follow-up periods are needed to assess the durability of the treatment response and monitor for any adverse outcomes, such as gastrointestinal disturbances, bleeding disorders, or allergic reactions, associated with EPO and vitamin E supplementation.

Our study has several limitations. First, the generalizability of our findings may be limited by the inclusion of only pre-menopausal women with cyclical mastalgia. The efficacy of EPO and vitamin E in other patient populations, such as postmenopausal women or those with non-cyclical breast pain, remains unclear and warrants further investigation. Second, adherence to treatment regimens may vary among participants, potentially influencing the treatment outcomes. Future studies incorporating objective measures of adherence, such as serum biomarkers or pill counts, could provide a more accurate assessment of treatment compliance.

The results of this study have valuable clinical implications for the management of cyclical mastalgia in premenopausal women. First, the findings suggest that combination therapy with EPO and vitamin E may provide superior relief from breast pain compared to individual treatments or placebo. This finding implies that clinicians should consider prescribing combination therapy as a potential first-line option for patients with cyclical mastalgia who do not respond adequately to monotherapy or conservative measures. By offering a synergistic effect, the combination of EPO and vitamin E may target multiple pathways involved in the pathophysiology of breast pain, resulting in more comprehensive symptom relief. Moreover, this study underscores the potential efficacy of EPO and vitamin E as standalone treatments for cyclical mastalgia. While combination therapy showed the most significant reduction in breast pain severity, both EPO and vitamin E alone demonstrated modest improvements compared with placebo. This suggests that these treatments may have independent analgesic effects and could be considered alternative treatment options, particularly for patients who cannot tolerate combination therapy or prefer simpler regimens. Clinicians can tailor treatment plans according to individual patient needs, taking into account factors such as treatment preferences, comorbidities, and potential drug interactions.

Additionally, the minimal adverse effects associated with EPO and vitamin E in this study suggest that both treatments are well-tolerated and safe for use in premenopausal women with cyclical mastalgia. This is reassuring for clinicians and patients alike because concerns about potential side effects may deter individuals from initiating or adhering to treatment. By emphasizing the favorable safety profiles of EPO and vitamin E, clinicians can help alleviate patient concerns and facilitate informed decision-making regarding treatment options. Furthermore, the high treatment adherence observed across all groups underscores the importance of patient education and counseling in promoting treatment compliance. Effective communication regarding the expected benefits, potential side effects, and proper administration of medications can enhance patients’ understanding and engagement in their care. Clinicians should take a proactive approach to patient education, provide clear instructions, and address any questions or concerns that may arise throughout the course of treatment.

Finally, while this study provides valuable insights into the efficacy of EPO, vitamin E, and their combination in managing cyclical mastalgia, further research is needed to refine treatment strategies and optimize outcomes. Future studies should explore the long-term effects of treatment beyond the six-month duration of this trial and investigate the potential mechanisms underlying the observed therapeutic benefits. By advancing our understanding of the pathophysiology of cyclical mastalgia and identifying novel therapeutic targets, future research can contribute to the development of more effective and personalized treatment approaches for this common and distressing condition.

## Conclusions

In conclusion, our study provides compelling evidence of the efficacy of EPO and vitamin E, either alone or in combination, in alleviating breast pain associated with cyclical mastalgia. The synergistic effects observed with combination therapy underscore the potential benefits of multimodal approaches for managing this challenging condition. While further research is needed to elucidate the optimal dosage, duration of treatment, and long-term safety profile of EPO and vitamin E supplementation, our findings offer promising insights into the management of cyclical mastalgia and highlight the importance of personalized, integrative approaches in addressing the complex needs of affected individuals.
